# Cervical Myelopathy and Peripheral Polyneuropathy in a 26-Year-Old Female Following Bariatric Surgery

**DOI:** 10.7759/cureus.15759

**Published:** 2021-06-19

**Authors:** Akash Jindal, Nicholas K Donohue, Christopher White

**Affiliations:** 1 Physical Medicine and Rehabilitation, Medical College of Wisconsin, Wauwatosa, USA

**Keywords:** sleeve gastrectomy, thiamine deficiency, copper deficiency, peripheral polyneuropathy, obesity treatment

## Abstract

The frequency, clinical course, and prognosis of the neurological sequelae following bariatric surgery remain obscure and continue to be a subject of medical research. We present the case of a 26-year-old female who underwent sleeve gastrectomy for the treatment of obesity and demonstrated progressive neurological deficits within months of her procedure. Extensive testing revealed very low thiamine and copper levels, peripheral polyneuropathy, and spinal cord lesions on imaging. She was treated with intravenous copper and thiamine and was admitted to the rehabilitation unit. Eighteen months following her admission, she achieved complete recovery.

Previous studies have reported neurological complications following bariatric surgery in 1.1-8.6% of cases. Some of the most common nutritional deficiencies involve copper, iron, calcium, magnesium, and vitamins B1, B12, D, and E. Patients may experience central and peripheral neurological deficits following bariatric surgery. Fortunately, a path to recovery exists and it involves both pharmacological and rehabilitative treatment.

## Introduction

Obesity is a chronic disease and a major contributor to poor health globally. The prevalence of severe obesity [body mass index (BMI) >40 kg/m^2^] reportedly increased from 5.7 to 9.2% between 2007 and 2018 [1]. While typical conservative approaches to the treatment of obesity include lifestyle adjustments in diet and exercise, more and more patients are turning to bariatric surgery as a more definitive treatment. One such surgical procedure is sleeve gastrectomy, in which approximately 75% of the stomach is surgically removed, reducing the remaining stomach to a tubular "sleeve" [2]. Most patients report significant weight loss following this surgery, and up to 60% weight loss has been reported two years following sleeve gastrectomy [3]. Central and peripheral neurologic complications following bariatric surgery have been documented infrequently, with some studies reporting a neurologic complication rate ranging from 1 to 9% [4-6]. In this report, we present a case of severe central and peripheral neuropathy following sleeve gastrectomy; we also describe the patient’s entire clinical course from symptom onset to the completion of her rehabilitation course over 18 months later.

## Case presentation

The patient was a 26-year-old female with a past medical history of severe obesity who had attempted multiple conservative approaches for weight loss but eventually underwent a laparoscopic sleeve gastrectomy at an outside hospital. Prior to the surgery, her BMI had been 55.2. Three months after the surgery, she lost almost 75 lbs and her BMI was down to 40.1. Following this loss, she began to experience severe episodes of nausea, vomiting, and significantly reduced appetite. A laboratory workup and CT scan of her abdomen were unrevealing, and she subsequently underwent an esophagogastroduodenoscopy (EGD), which showed a gastro-jejunal stricture that was balloon dilated.

**Figure 1:**
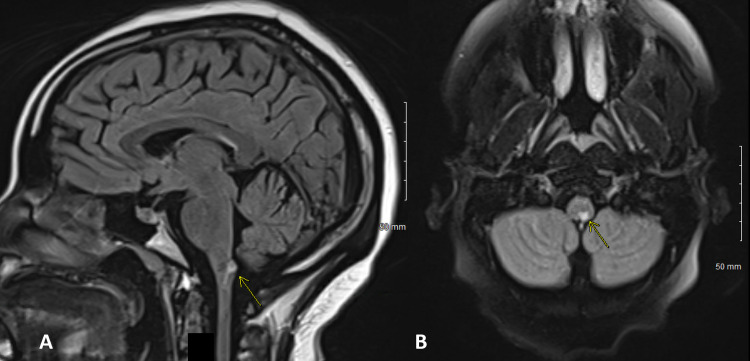
MRI FLAIR images in the sagittal (A) and axial (B) planes demonstrating the lesion at the cervical-medullary junction (arrows) MRI: magnetic resonance imaging; FLAIR: fluid-attenuated inversion recovery

**Figure 2:**
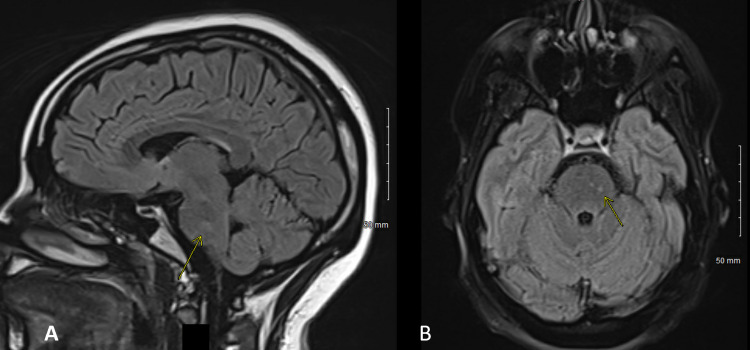
MRI FLAIR images in the sagittal (A) and axial (B) planes demonstrating lesions in the pons (arrows) MRI: magnetic resonance imaging; FLAIR: fluid-attenuated inversion recovery

Four months following her bariatric surgery, the patient presented to the emergency department (ED) of a quaternary academic medical center with a two-day history of severe headaches, blurred vision, unsteady gait, and reduced sensation in her feet. MRI brain showed three enhancing lesions in the pons and cervical-medullary junction on fluid-attenuated inversion recovery (FLAIR) sequences (Figures 1, 2). A lumbar puncture was performed and cerebrospinal fluid (CSF) studies were normal. Serum studies were significant for low thiamine (vitamin B1) levels. She underwent a five-day course of intravenous (IV) Solu-Medrol with a mild improvement in symptoms. No clear diagnosis was made at the time, and the patient was discharged home and underwent weekly IV infusions of thiamine. She was followed up closely by Neurology, who re-admitted her approximately one month later for new saccadic nystagmus, increased lower extremity weakness, and progressive hypoesthesia in her bilateral feet. Repeat MRI brain revealed findings similar to her previous admission; repeat CSF studies were also normal, and serum studies were significant for persistently low thiamine levels in addition to very low copper levels. Given her lack of improvement with previous thiamine infusions and progressive symptoms, she underwent plasma exchange and continued IV replacement of vitamins and minerals. She was also initiated on gabapentin for neuropathic pain in her bilateral hands and feet.

On day three of her admission, the patient underwent electrodiagnostic testing. Upper and lower extremity sensory nerve conduction testing showed no response diffusely. Motor conduction testing was significant for low amplitudes and mildly slowed conduction velocities diffusely. Needle electromyography (EMG) was largely normal with few, high-amplitude fibrillations and mild polyphasia in the tibialis anterior and vastus medialis. Overall, the testing was suggestive of an acute, length-dependent, symmetric, sensorimotor polyneuropathy of a primarily axonal type. She was admitted to the inpatient rehabilitation service on day 10 of her acute admission. After three weeks of rehabilitation focused on lower extremity strengthening and balance, she was discharged home in a wheelchair, requiring some assistance for activities of daily living (ADL). Compared to her physical exam at admission, her upper extremity strength had increased but her lower extremity testing showed a 2/5 strength in bilateral dorsiflexion and great toe extension, with the remainder of bilateral lower extremity testing showing a 4/5 strength. She was discharged on a regimen of 800 mg gabapentin four times per day to continue to address her neuropathic pain in a stocking-glove distribution.

Over the next six months, the patient continued to be followed up closely by her neurologist and physiatrist. She received intermittent IV infusions of thiamine and copper but was eventually placed on a stable oral replacement regimen. She consistently participated in outpatient therapies but was still using a wheelchair for mobility six months after her discharge. A repeat MRI brain showed stable pontine and upper cervical enhancing foci with no new lesions. At her 12-month follow-up appointment, she reported that she had been walking short distances and only used a wheelchair for longer distances. This was primarily due to balance issues and not strength. Her physical exam at the time revealed a 5/5 strength diffusely for the first time since her initial symptoms. At her 18-month follow-up appointment, she was no longer using an assistive device for mobility and had completely weaned off of gabapentin without significant pain in her hands and feet. She was eager to undergo a driving evaluation and was also referred to vocational rehabilitation, as she was hopeful of returning to work.

## Discussion

This case discusses the potential neurological complications following bariatric surgery. Although considered to be rare, the incidences of such complications have been reported in the literature and have shown a varying pattern. Tabbara et al. performed a retrospective review of 592 patients who had undergone bariatric surgery and reported neurological symptoms in only 1.18% [4]. Abarbanel et al. reviewed the charts of 500 patients who had undergone bariatric surgery [5] and found that 4.6% of their patients showed neurological complications that developed 3-20 months after surgery [5]. Thaisetthawatkul et al. reviewed charts of 556 patients who had undergone bariatric surgery at the Mayo Clinic from 1980-2003, and they reported that 8.6% of their patients experienced neurological complications, primarily peripheral neuropathies (74%) [6]. The primary cause of these complications is nutritional deficiency, which can lead to a broad spectrum of other disorders such as Wernicke encephalopathy, myelopathies, ataxia, plexopathies, peripheral neuropathies, cranial nerve palsies, and seizures [7]. Some of the most common mineral deficiencies following bariatric surgery involve copper, iron, calcium, magnesium, and vitamins B1, B12, D, and E [7]. Many patients who develop nutritional deficiencies and subsequent neurological deficits have had a history of repeated vomiting and poor oral intake, which can exacerbate these deficiencies [8]. Rapid and significant weight loss has also been associated with neurological complications [9]. In our patient, the primary deficiencies that were identified were vitamin B1 and copper. She also demonstrated a significant weight loss of almost 75 lbs in the first three months after her surgery, which was associated with multiple episodes of vomiting months after the procedure.

Along with altering mitochondrial function and impairing oxidative metabolism, thiamine deficiency can cause selective neuronal death by diminishing thiamine-dependent enzymes [7]. A lack of thiamine has been shown to cause peripheral neuropathy, nystagmus, ataxia, and encephalopathy [7]. Although our patient was not encephalopathic at any point in her course, she did present with the other signs and symptoms that were consistent with bariatric beriberi. The neuropathy that typically develops with thiamine deficiency affects the lower limbs and can variably affect the sensory and motor nerves. The results of our patient’s electrodiagnostic testing were very similar to those presented elsewhere in the context of thiamine deficiency, namely a peripheral polyneuropathy that is length-dependent and axonal with markedly reduced amplitudes of both compound motor action potential and sensory nerve action potentials [7,9]. Additionally, thiamine deficiency has shown characteristic central nervous findings on MRI, specifically hyperintense signal abnormalities on FLAIR or T2-weighted images in the dorsomedial thalamic nuclei, periaqueductal gray matter, mammillary bodies, and cervical spine [7]. As mentioned above, our patient’s MRI showed three enhancing lesions in the pons and cervical-medullary junction.

Although less commonly reported, deficiencies in serum copper following bariatric surgery have been known to occur [10]. Copper is an essential cofactor in many enzymatic reactions that are key to various systems in the body, including the structure and function of the neurological system. Typical clinical symptoms associated with copper deficiency include extremity paresthesias, impaired vibratory sensation and proprioception, and gait abnormalities [11]. Griffith et al. have presented two cases, which were followed prospectively, where patients presented with severe gait abnormality and neurological deficits to their lower extremities years after bariatric surgery [12]. While our patient experienced significant improvement in symptoms after 18 months of treatment, the patients presented by Griffith et al. only had mild neurological improvements even after IV and oral repletion of copper [12]. Tan et al. have reported the case of a copper-deficient patient who presented with severe ataxia and lower extremity paresthesias and weakness following sleeve gastrectomy [13]. The patient was treated with IV and subsequent oral supplementation with improvement in the paresthesias but not the weakness. Given that copper deficiency can lead to irreversible impairments, it is crucial to diagnose and treat these patients with supplementation in a timely manner. Many patients with copper deficiency can also present with hematological abnormalities such as hypochromic anemia, neutropenia, and leukopenia [14]. If these changes are seen following bariatric surgery, concerns for copper deficiency may be reasonable. The patient presented in this case demonstrated no hematological changes.

## Conclusions

We discussed the case of a patient who was found to have central and peripheral neurological deficits following bariatric surgery due to deficiencies in thiamine and copper. Fortunately, this young patient made a complete recovery after 18 months of both pharmacological and rehabilitative treatment. Given the significant increase in the frequency of bariatric surgery as a treatment for obesity in recent years, it is important for medical providers to become familiar with the potential neurological sequelae of these surgeries. This case not only presents the diagnosis and acute treatment of these postoperative neurological deficits but also provides an example of a hopeful prognosis.
